# Inverse MDS: Inferring Dissimilarity Structure from Multiple Item Arrangements

**DOI:** 10.3389/fpsyg.2012.00245

**Published:** 2012-07-25

**Authors:** Nikolaus Kriegeskorte, Marieke Mur

**Affiliations:** ^1^Cognition and Brain Sciences Unit, Medical Research CouncilCambridge, UK; ^2^Maastricht UniversityMaastricht, Netherlands

**Keywords:** multidimensional scaling, representation, representational similarity analysis, similarity, similarity judgment

## Abstract

The pairwise dissimilarities of a set of items can be intuitively visualized by a 2D arrangement of the items, in which the distances reflect the dissimilarities. Such an arrangement can be obtained by multidimensional scaling (MDS). We propose a method for the inverse process: inferring the pairwise dissimilarities from multiple 2D arrangements of items. Perceptual dissimilarities are classically measured using pairwise dissimilarity judgments. However, alternative methods including free sorting and 2D arrangements have previously been proposed. The present proposal is novel (a) in that the dissimilarity matrix is estimated by “inverse MDS” based on multiple arrangements of item subsets, and (b) in that the subsets are designed by an adaptive algorithm that aims to provide optimal evidence for the dissimilarity estimates. The subject arranges the items (represented as icons on a computer screen) by means of mouse drag-and-drop operations. The multi-arrangement method can be construed as a generalization of simpler methods: It reduces to pairwise dissimilarity judgments if each arrangement contains only two items, and to free sorting if the items are categorically arranged into discrete piles. Multi-arrangement combines the advantages of these methods. It is efficient (because the subject communicates many dissimilarity judgments with each mouse drag), psychologically attractive (because dissimilarities are judged in context), and can characterize continuous high-dimensional dissimilarity structures. We present two procedures for estimating the dissimilarity matrix: a simple weighted-aligned-average of the partial dissimilarity matrices and a computationally intensive algorithm, which estimates the dissimilarity matrix by iteratively minimizing the error of MDS-predictions of the subject’s arrangements. The Matlab code for interactive arrangement and dissimilarity estimation is available from the authors upon request.

## Introduction

Mental representations can be conceptualized as representations of the similarities between the perceived objects (Edelman, 1998). Judgments of the similarities among a set of objects provide important evidence about a subject’s mental representation of the objects and their relationships. A natural way of explaining dissimilarity judgments is by assuming a geometric model of the mental representation (e.g., Carnap, 1928; Coombs, 1954; Shepard, 1958; Torgerson, 1958, 1965). In a geometric model, dissimilarities are interpreted as distances in a multidimensional space (and similarities as proximities). The dimensions of the space could be objective or subjective properties.

This suggests that we could ask subjects to judge item properties instead of dissimilarities. We could then assume the geometric model and compute the dissimilarities as distances in the space spanned by the properties. However, this would require prespecification of the properties and their relative weights. In dissimilarity judgments, by contrast, the subject must choose and weight any underlying properties – consciously or unconsciously. Dissimilarity judgments therefore promise to provide evidence of the subject’s mental representation that is not biased by item properties prespecified by the researcher. (The disadvantage is that the properties underlying the judgments are not explicitly known, although they may be inferred.)

Geometric models of similarity have been criticized (e.g., Goodman, 1972; Tversky, 1977; Goldstone et al., 1997) in the light of empirical demonstrations that dissimilarity judgments can be context dependent (e.g., a cat and a dog might appear more similar when a bird is added to the set), intransitive [e.g., dissimilarity(X,A) < dissimilarity(X,B) < dissimilarity(X,C), but dissimilarity(X,C) < dissimilarity(X,A)], and asymmetric (e.g., Korea may be judged as more similar to China, than China to Korea). However, more sophisticated versions of the geometric model (for an overall framework, see Gärdenfors, 2004) can account for these anomalies (Decock and Douven, 2011). The anomalies are accounted for by allowing flexible selection and weighting of the dimensions of the space. Different tasks may be associated with different weightings of the dimensions (e.g., looking for food may render a different set of attributes salient than looking for shelter). Dimension weighting may also be affected by item set context and by the order of presentation of the items, accounting for the effect of these variables on dissimilarity judgments.

The geometric model is attractive (1) for its natural treatment of the continuous variation of multiple properties (in contrast to binary feature set accounts, e.g., Tversky, 1977), (2) for its derivation of dissimilarity predictions from property representations, and (3) for its relationship to distributed representations of objects in computational models (e.g., McClelland and Rogers, 2003) and in the brain (e.g., Edelman et al., 1998; Haxby et al., 2001; Kriegeskorte et al., 2008b). Here we assume a geometric model and address a practical research problem: How to efficiently acquire dissimilarity judgments. We propose having the subject perform multiple arrangements of item subsets adaptively designed for optimal measurement efficiency and to estimate the representational dissimilarity matrix (RDM) by combining the evidence from the subset arrangements.

## Methods for Acquiring Dissimilarity Data from Subjects

To motivate the multi-arrangement method, we now briefly review different ways of acquiring dissimilarity data from subjects. Table 1 also summarizes the pros and cons of these methods.

**Table 1:** **Different behavioral methods for acquiring dissimilarities**.

	Description	Pros	Cons
**(1) Pairwise similarity judgment**	Each pair of items is presented in isolation and the subject rates the dissimilarity on a scale	• Each pair is independently rated (this is a pro, if set context is thought to distort judgments or a con, if set context is thought to anchor and inform judgments)	• Slow: (*n*^2^ − *n*)/2 separate judgments* required, thus only feasible for small item sets • Interpretation of the dissimilarity scale may drift as previous judgments are not visible for comparison
**(2) Free sorting**	The subject sorts the items into a freely chosen number of piles (i.e., categories)	• Quick: requires only *n* placements*, thus has essentially linear time complexity (neglecting the time taken to decide the categories), thus feasible for large item sets	• Gives only binary dissimilarities (same pile, different pile) for a single-subject • Category definition might be dominated by the first items and might drift if piles are perceived to be represented by the item on top
**(3) Single arrangement**	The subject arranges the items in 2D with the distances taken to reflect the dissimilarities	• Relatively quick: each placement of an item communicates multiple dissimilarity judgments (superlinear, but subquadratic time complexity) • The relationships of multiple pairs are considered in context	• Restriction to 2D prevents communication of higher-dimensional dissimilarity structures
**(4) Multi-arrangement** (proposed method)	A generalization of (1), (2), and (3), in which multiple item subsets are arranged in a low-dimensional (e.g., 2D) space and the dissimilarity structure is inferred from the redundant distance information	• Includes methods (1)–(3) as special cases, so cannot do worse • Enables us to quickly acquire judgments reflecting higher-dimensional dissimilarity structures • Anytime behavior: process can be terminated anytime after a first trial containing all items (=single arrangement) • Addresses the cons of methods (1), (2), and (3)	• Requires a method for constructing subsets (which may involve assumptions that affect the results) • Requires a method for estimating the dissimilarity structure from multiple item-subset arrangements (which may involve assumptions that affect the results)
**(5) Arrangement of pairs by dissimilarity** (proposed here for comparison purposes)	Each item pair is represented by a visual icon, and the subject arranges the icons along a 1D dissimilarity scale	• Dissimilarities are judged in the context of all other pairwise dissimilarities • Each pair is independently rated	• Time-intensive: (*n*^2^ − *n*)/2 separate judgments* required • Space-intensive: (*n*^2^ − *n*)/2 pair icons need to fit along the scale • Only feasible for small item sets for the above reasons
**(6) Implicit measures: confusions and discrimination times** (not discussed here in detail)	Subject performs a task requiring discrimination among the items. If two items are more frequently confused or take longer to discriminate, they are considered more similar	• Reflects perceptual representations that might not be reflected in explicit judgments	• Slow: (*n*^2^ − *n*)/2 separate trials required, thus only feasible for small item sets • Not informative about explicit judgments

*Where *n* is the number of items.

### Pairwise dissimilarity judgments

In pairwise dissimilarity judgments (e.g., Cortese and Dyre, 1996), the subject is presented with one pair of items at a time and rates the dissimilarity (or similarity) of the two items. Such a rating is performed for each pair of items. This straightforward technique requires (*n*^2^ − *n*)/2 trials (one per pair), where *n* is the number of items. For 10 items, thus, we require 45 trials, for 50 items 1225 trials, and for 100 items 4950 trials. Because of the quadratic growth of the time requirement, this method is not feasible for large sets of items. Independent pair judgment places no constraints on the relationships between the judgments. Under a geometric model, this allows us to capture dissimilarity structures of arbitrary dimensionality. In addition, it allows us to capture judgment data inconsistent with a geometric model. A potential disadvantage of separate judgment of each pair is that the subject’s interpretation of the different degrees of dissimilarity might be unstable across a long session of judgments, because previous judgments are not visible for comparison.

### Free sorting

In free sorting (e.g., Coxon, 1999), the subject sorts the items into a freely chosen number of piles (i.e., categories). Note that this is distinct from sorting the items into a sequence, where their place in the sequence corresponds to their rank on some property dimension. Free sorting enforces categorization, although the categories can be freely defined. The result of a single sorting is a binary dissimilarity matrix indicating for each pair of items, whether the two items were in the same pile or in different piles. The major advantage of this method is that it requires only *n* placements for *n* items, and thus has essentially linear time complexity if we neglect the time taken to decide the categories. This renders free sorting feasible for large item sets. The major disadvantage is that the method gives only binary dissimilarities (same pile, different pile) for a given sorting. If the subject chooses to use few piles, then more subtly dissimilar items are lumped together as though they were identical. If the subject chooses to use many piles, then subtly dissimilar items are represented in the same way as extremely dissimilar items. Another caveat is that the category definition might be strongly influenced by the first items if the subject defines the categories *ad hoc* as the sorting progresses. Moreover, the category definition might drift, if the subject perceives each pile to be represented by the item on top.

### Single arrangement

Similarities and dissimilarities can be intuitively captured by a physical arrangement of the items. Under a geometric model, we would assume such an arrangement to approximate the distances in the internal representational space. This suggests having the subject arrange the items in 2D, placing similar items close together and dissimilar items far apart, such that the distances can be interpreted as dissimilarities. (Note that the power of an arrangement to intuitively convey dissimilarities also motivates the dissimilarity visualization technique of multidimensional scaling (MDS, Torgerson, 1958; Shepard, 1962; Borg and Groenen, 2005.) The arrangement method for acquiring dissimilarity judgments has been described by Risvik et al. (1994) and Goldstone (1994). It is sometimes referred to as “projective mapping.” For early precursors, see Oppenheim (1966).

This method is quicker than pairwise judgment because each placement of an item communicates multiple dissimilarity judgments. An additional potentially attractive feature is that the relationships of multiple pairs are considered in context, as all items are always in view. Arrangement is superior to free sorting in that it enables the subject to convey continuously varying dissimilarities. This advantage will usually come at a cost: If the subject carefully considers the continuous pairwise dissimilarities in arranging the items, the process will take longer than free sorting. We expect a time complexity that is larger than that of free sorting, but smaller than that of pairwise judgment (superlinear, but subquadratic in the number of items). The major disadvantage of the single arrangement method is the restriction to 2D, which prevents communication of higher-dimensional dissimilarity structures.

### Multi-arrangement

The single arrangement method can be extended to multiple arrangements. For example, Goldstone (1994) had subjects arrange multiple random subsets of 20 out of 64 items. Here we refer to this approach as “multi-arrangement” and describe methods for adaptive design of the item subsets (so as to optimize measurement efficiency) and for combining the multiple arrangements into a single dissimilarity estimate (inverse MDS).

In the multi-arrangement method, the subject arranges multiple item subsets in a low-dimensional (e.g., 2D) space and the dissimilarity structure is inferred from the redundant distance information. This approach can be viewed as a generalization of methods (1), (2), and (3): It reduces to pairwise dissimilarity judgments if each arrangement contains only two items, and the arrangement consists merely in choosing a single distance. It reduces to free sorting if the items are arranged into discrete piles and the distances binarized into small distances (within pile) and large distances (between piles). It reduces to a single arrangement of all items if subset arrangements are omitted. For a given application, we have no reason to expect that the optimal method will be one of the special cases [methods (1)–(3)]. Multi-arrangement promises to combine the advantages of other methods to some extent.

Compared to pairwise judgments, multi-arrangement can be more efficient (because the subject communicates many dissimilarity judgments with each mouse drag) and psychologically attractive (because dissimilarities are judged in the context of a larger set). Compared to free sorting, multi-arrangement is suited for acquiring continuously varying dissimilarities (as opposed to binary dissimilarities corresponding to categorical structures). Compared to a single arrangement, multi-arrangement can recover dissimilarity structures whose dimensionality is greater than 2.

### Arrangement of pairs by dissimilarity

We propose an additional method here, mainly for validation purposes. In this method, each item *pair* is represented by a visual icon. The subject arranges the pair icons along a 1D dissimilarity scale. Note that this is not a special case of multi-arrangement, but it is closely related to pairwise judgment in that an independent dissimilarity judgment is communicated for each item pair. In contrast to pairwise judgment, however, the dissimilarities are judged in the context of all other pairwise dissimilarities. This promises a more precise judgment of the dissimilarities. Like pairwise judgment, the method is time-intensive because (*n*^2^ − *n*)/2 pair-icon placements are required. In addition, it is space-intensive as the (*n*^2^ − *n*)/2 pair icons need to fit along the scale. As a result, this method is only feasible for small item sets.

### Implicit dissimilarity measures: Confusion frequency and discrimination time

Perceptual dissimilarities can also be inferred from confusions (as more similar items are likely to be confused more frequently) or from reaction times in discrimination tasks (as more similar items are likely to take longer to discriminate). Like explicit pairwise judgments, these implicit techniques require a number of trials that grows quadratically with the number of items. Here we focus on explicit judgments, specifically method (4) and, for validation, methods (1) and (5).

## The Multi-Arrangement Method

### Behavioral method: 2D drag-and-drop object arrangements

Item arrangements could be performed on a table top using either the items themselves if they were small and movable (e.g., glasses of beer; Abdi and Valentin, 2007; Lelièvre et al., 2008) or physical symbols of the items (e.g., photos). The distances would then have to be measured for data analysis. In order to avoid the complications of physical arrangements, we use virtual arrangements performed on a computer screen by mouse drag-and-drop operations (Goldstone, 1994).

**Figure 1:**
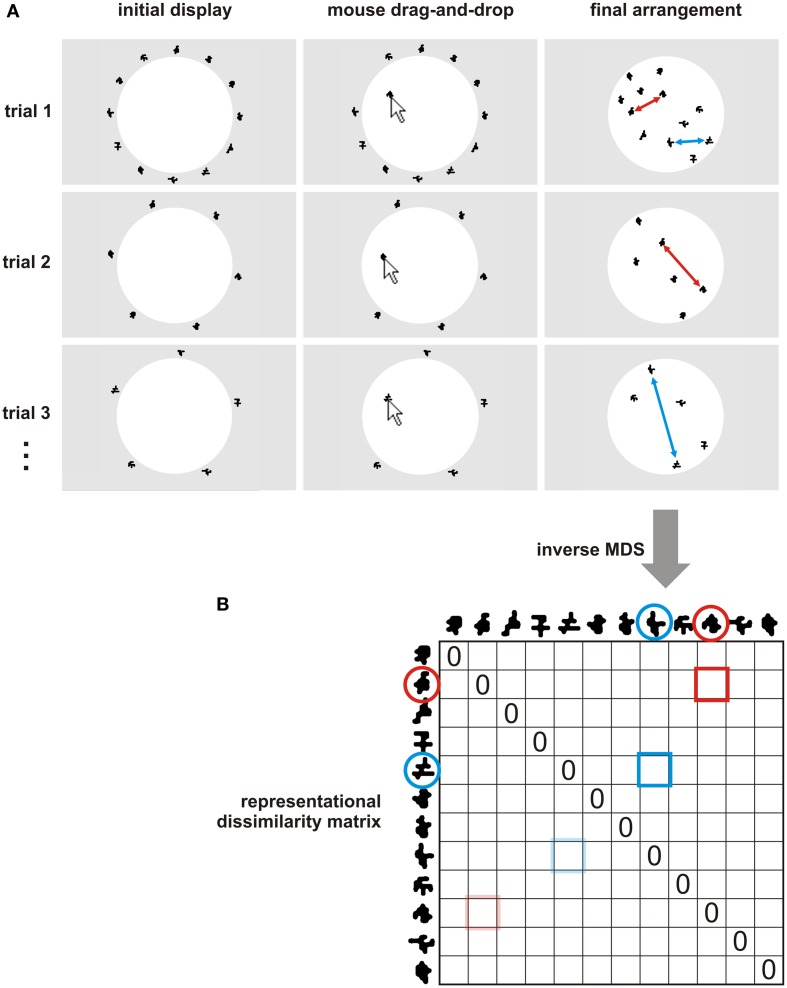
**Multi-arrangement method for acquiring a subject’s dissimilarity judgments**. **(A)** In the multi-arrangement method, the subject arranges the items (represented as icons on a computer screen) by means of mouse drag-and-drop operations. Perceived similarity is communicated by adjusting the distances between the objects: objects perceived as similar are placed together; objects perceived as dissimilar are placed apart. Multiple arrangements (trials) are performed for the entire set and for subsets of the items, so as to allow the subject to convey a higher-dimensional dissimilarity structure. Subsets are constructed by an adaptive algorithm (Multi-Arrangement by Lift-the-Weakest Algorithm for Adaptive Design of Item Subsets in Appendix), which is designed to efficiently sample the evidence for estimation of the representational dissimilarities. **(B)** The evidence from the multiple 2D arrangements is statistically combined to obtain the estimate of the representational dissimilarity matrix (RDM). See Figure 3 and Estimating the High-Dimensional Dissimilarity Matrix from Multiple Item-Subset Arrangements in Appendix for algorithms. The RDM contains a dissimilarity estimate for each pair of items and is symmetric about a diagonal of zeros. Two example item pairs are shown in red and blue. Their single-trial dissimilarity estimates (red and blue double arrows in **(A)**) are combined into a single dissimilarity estimate. Mirror-symmetric entries are indicated by transparent colors.

The icons are to be arranged in a designated screen area, which we call the “arena.” Initially, the items are displayed outside the arena, in an area we call the “seating.” In one implementation, the arena is circular and the seating surrounds the arena (Figure 1A). In another implementation, the arena and the seating are rectangular and placed side by side or one above the other. Within the seating, the items can initially be presented in either a predefined or a random arrangement.

The subject can move any item by “dragging” it (i.e., clicking on it with the left mouse button and moving the mouse while keeping the button pressed). In this way, the subject initially arranges all items. To indicate that the arrangement is final, the subject clicks on a button marked “Done.” The distance matrix of the initial arrangement of all items provides an initial estimate of the RDM. After the initial arrangement, the subject proceeds to arrange subsets of items. We refer to each arrangement as a “trial.” The process can be terminated after any trial (e.g., when the time the subject can spend on the task has elapsed). All pairwise dissimilarities are then estimated from the available arrangements of item subsets.

### Adaptive design of the item-subset for each arrangement trial

For each trial, we need to decide what item-subset to present to the subject. One approach is to have the subject arrange a random item-subset of a prespecified size on each trial (Goldstone, 1994). However, random subsets have several drawbacks. First, we cannot be sure that each item is sampled at all and that each item pair appears together in a subset at some point for its dissimilarity to be directly judged by the subject. This can be mended by adding appropriate constraints in defining the subsets. Second, and more importantly, random subsets will tend to be globally distributed throughout the item set. As a result, the context of each arrangement will be essentially that of the entire set for larger item sets. (Larger item subsets are preferable, because they provide more dissimilarity measurements per placed item.)

Consider the case of a set of object images spanning a wide range of categories, including animates and inanimates and, within the animates, faces, and bodies (Kriegeskorte et al., 2008b). The faces might form a subcluster in the subject’s mental representation, suggesting a presentation of a trial including only faces. This would allow the subject to use the entire arena for the faces, giving us good evidence of their relative dissimilarities. Using random subsets, however, the faces are unlikely to appear together in a trial in the absence of very different (e.g., inanimate) objects. As a result, whatever faces are present will end up in a corner of the arena and so close together that their relative distances cannot be reliably distinguished from placement error.

The proposed method, by contrast, starts with an arrangement of the entire set, and presents clusters of similar items together on subsequent trials. Item-subset design is *adaptive* in the sense that it depends, for each trial, on the current estimate of the RDM. We instruct the subject to use the entire arena to arrange the subset presented on a given trial. Because the subject is instructed to “zoom in” on the current subset, there is no fixed relationship of screen distance and dissimilarity that holds across trials. Instead, we assume only that the relative screen distances reflect the relative dissimilarities on each trial (i.e., the on-screen distance *ratios* reflect the dissimilarity *ratios*).

We start with a trial that includes all items. This initial arrangement provides a rapid estimate of the entire RDM. However, this estimate has two deficiencies. First, it is restricted to 2D. This motivates subsequent trials with item subsets. Second, assuming that each on-screen placement of an item is affected by placement error (drawn from the same distribution, e.g., a 2D Gaussian), the dissimilarity signal-to-noise ratio will be lower for two items judged as similar and thus placed close together (small dissimilarity signal) than for two items judged as dissimilar and thus placed far apart on the screen (large dissimilarity signal). This motivates selective re-sampling of item pairs placed close together on the first trial.

The method by which trial efficiency is optimized is precisely defined in Section “Multi-Arrangement by Lift-the-Weakest Algorithm for Adaptive Design of Item Subsets” in Appendix. Briefly, the goal of our method is to gather roughly equal amounts of evidence for each dissimilarity. This is achieved by maintaining a record of the amount of evidence already gathered for each dissimilarity and designing subsequent item subsets such that they provide evidence for those dissimilarities, for which we have the weakest evidence. The continual focus on the dissimilarities, for which we have the weakest evidence, lends our method its name, “lift-the-weakest” algorithm. The algorithm rapidly provides a rough estimate of the RDM and then gathers the most urgently needed evidence on each trial. As a result, the algorithm has “anytime behavior,” in the sense that it can be terminated after any trial (although the quality of the estimate will increase as more data is gathered).

### Estimating dissimilarities from multiple arrangements: Average of scaled-to-match arrangements and inverse MDS

Each trial provides a partial RDM. This raises the question how the multiple partial matrices should be combined to give a single estimate of the entire RDM. In Section “Estimating the High-Dimensional Dissimilarity Matrix from Multiple Item-Subset Arrangements” in Appendix, we define two methods for obtaining such an estimate. The first method estimates each dissimilarity as a weighted average of the distances in the arrangements in which the item pair was included. Each arrangement is first scaled to adjust for the fact that the subject zooms in on the item-subset presented on each trial. The weighted average is computed with iterative rescaling as described in Section “Weighted Average of Iteratively Scaled-to-Match Subset Dissimilarity Matrices” in Appendix.

The second, more sophisticated method estimates the RDM by inverse MDS. Recall that MDS takes a distance matrix for *n* items as its input and gives an arrangement of the items in a low-dimensional space (e.g., 2D, for visualization), such that the new distances optimally approximate the original distances. Inverse MDS, then, should proceed in the opposite direction: from a low-dimensional arrangement to the original high-dimensional distance matrix. We can get one solution by simply measuring the distances between the points in the low-dimensional arrangement. (Whereas approximating the distances from a higher-dimensional arrangement in a low-dimensional arrangement, i.e., MDS, is difficult, the opposite direction is trivial.) However, there will typically by many solutions, i.e., many high-dimensional arrangements optimally represented by a given low-dimensional arrangement. We could ask of inverse MDS to return the *set* of all distance matrices, whose MDS solution is the low-dimensional arrangement given as input (De Leeuw and Groenen, 1997).

Motivated by the practical problem of inferring a distance matrix from multi-arrangement data, we use a more general conceptualization here: Inverse MDS is given a *set* of low-dimensional arrangements of item subsets as input and the goal is to infer the underlying high-dimensional distance matrix. Each arrangement adds constraints to the solution, but there might still not be a unique solution. Moreover the arrangement data is somewhat affected by error. We describe an algorithm that can be used improve an initial estimate of the RDM by predicting the arrangements expected for each item-subset (using MDS with metric stress as the criterion) and then iteratively adjusting the estimate of the RDM, so as to drive down the error of the predicted arrangements. The algorithm is illustrated in Figure 3 and properly defined in Section “Inverse Multidimensional Scaling by Iterative Reduction of Arrangement Prediction Error” in Appendix.

## Validation by Comparison to Conventional Behavioral Methods

**Figure 2:**
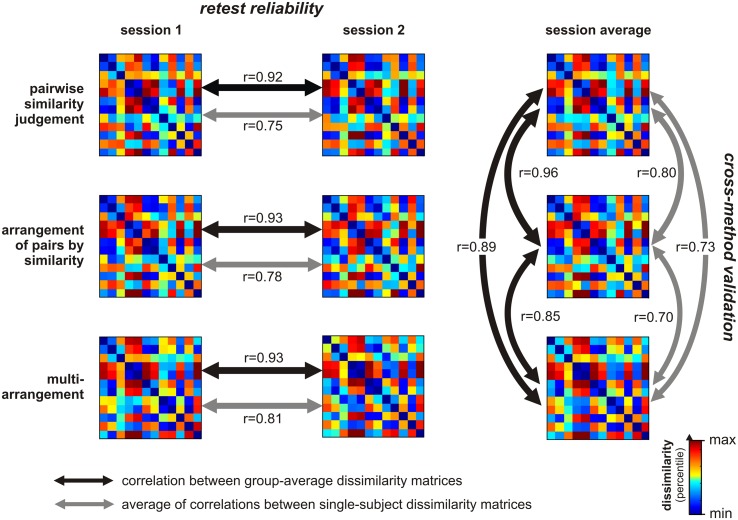
**Validation of multi-arrangement method**. We validated the multi-arrangement method by comparison to pairwise similarity judgments and arrangement of pairs by similarity, for a set of 12 shapes (Figure 1). We also computed the test-retest reliability for each method. Shown are group-average RDMs containing pairwise dissimilarity estimates acquired for each method and session. The RDMs were separately histogram-equalized for easier comparison. Across-methods consistency and test-retest reliability were computed by correlating the RDMs across methods and sessions, respectively. We performed two variants of this analysis that differed in the way the data were combined across subjects: we either averaged the single-subject data at the level of the RDMs (black double arrows) or at the level of the correlation coefficients (gray double arrows). Each arrow’s line thickness is proportional to the Spearman correlation coefficient it represents. Condition-label randomization tests on the black-arrow correlation coefficients showed that they are all significantly positive (*p* < 0.0001 for each comparison). These findings indicate that the test-retest reliability of all three methods is high, and suggest that multi-arrangement is a valid method for measuring perceived similarity.

We have validated our implementation of the multi-arrangement method for a set of 12 shapes by comparison to pairwise dissimilarity judgments and arrangement of pairs by dissimilarity (Figures 1 and 2). We also computed the test-retest reliability of the multi-arrangement method and the conventional methods.

### Subjects

Four healthy human volunteers (female; mean age: 29 years, age range: 27–34 years) participated in the validation experiment. Before participating, the subjects received information about the experimental procedure and gave their written informed consent for participating. The experiment was conducted in accordance with the guidelines of the Ethics Committee of the Faculty of Psychology and Neuroscience, Maastricht University.

### Experimental procedure

The experiment consisted of two 45-min sessions on separate days (2–4 days between the two sessions). During each session, subjects performed dissimilarity judgments on the 12 shapes using the following three methods: (1) multi-arrangement, (2) pairwise dissimilarity judgments, and (3) arrangement of pairs by dissimilarity. All three methods were implemented in Matlab. The order of the methods was counterbalanced across subjects and sessions. In each method, subjects communicated their judgments by arranging shapes or shape pairs on a computer screen by mouse drag-and-drop.

(1) The multi-arrangement method used a circular arena as shown in Figure 1A (white disk). On each trial, the shapes were initially presented in a circular arrangement around the arena, placed at regular angular intervals in random order. Subjects were instructed to arrange the shapes according to their similarity (similar shapes together, dissimilar shapes apart). Acquisition was terminated after all pairwise dissimilarities were lifted above a certain evidence weight threshold. We used an evidence weight threshold of 0.5 (and an evidence-utility exponent of 10). The RDMs were estimated by the average of scaled-to-match partial RDMs.
(2) The pairwise dissimilarity judgment method used a rectangular arena (white horizontal bar). On each trial, two shapes were shown: one in the left corner of the bar and one below the midpoint of the bar. Subjects were instructed to place the bottom shape inside the white bar, such that its distance to the reference shape along the horizontal axis reflected the perceived dissimilarity between the two shapes (similar shapes together, dissimilar shapes apart). Shape pairs were presented in random order. For each pair, the reference shape was assigned randomly. Acquisition was terminated when each possible shape pair had been judged once.
(3) The arrangement of pairs by dissimilarity method used a rectangular arena (white vertical bar) covering the left side of the computer screen. The right side of the screen displayed all possible shape pairs, presented in random order from top to bottom. Subjects were instructed to move the shape pairs to the arena and arrange them along the vertical axis according to their dissimilarity (pairs of similar shapes at the top, pairs of dissimilar shapes at the bottom). The arrangement was terminated when the subject had arranged all shape pairs and clicked on the “Done” button.

### Results

Each of the three similarity judgment methods yielded an RDM (Figure 1B) containing a dissimilarity estimate for each possible pair of shapes. Test-retest reliability was assessed by correlating the RDMs across the two sessions (different days). Across-methods consistency (as a measure of validity) was assessed by correlating the RDMs across-methods. Statistical inference on the Spearman correlation coefficients was performed using a condition-label randomization test (Kriegeskorte et al., 2008a). Figure 2 shows that the multi-arrangement method has a high test-retest reliability (*r* = 0.93, *p* < 0.0001). The other two methods had similarly high test-retest reliabilities (0.92 < *r* < 0.93, *p* < 0.0001). In addition, the RDMs acquired with the multi-arrangement method correlate well with those acquired using the other similarity judgment methods (0.85 < *r* < 0.89, *p* < 0.0001). The other two methods also correlate highly with each other (0.96, *p* < 0.0001). Overall, these analyses suggest that all three methods work well and give consistent results.

## Discussion

The contribution of this paper is twofold. First, practically, we offer a working method for efficient acquisition of dissimilarity judgments. This is important because it places larger sets of items (e.g., >100 items, >(100^2^ − 100)/2 = 4950 pairs) within realistic reach of behavioral studies. The Matlab code for the interactive arrangement and analysis is available from the authors upon request. Second, theoretically, we explore the concept of inverse MDS as inference of dissimilarities from multiple partial arrangements and describe an iterative algorithm for finding solutions.

### An efficient way of acquiring dissimilarity judgments

The pairwise dissimilarities of a set of items are commonly acquired using pairwise dissimilarity judgments or free sorting. Neither of these methods is well suited for acquiring continuous dissimilarity judgments for large numbers of items. The multi-arrangement method can handle this scenario. This approach is more efficient than pairwise dissimilarity judgments because each placement communicates multiple dissimilarities. The efficiency is further increased by adaptive design of the item-subset presented on each trial. Unlike free sorting, multi-arrangement provides continuous dissimilarity estimates. Multi-arrangement is intuitive and more strongly emphasizes the context of the items than either pairwise judgment or free sorting. Through multiple subset arrangements subjects can convey dissimilarity structures exceeding the 2D available for a single arrangement.

### Interpretation of the dissimilarity judgments

Multi-arrangement involves arrangement of multiple item subsets. The context, in which dissimilarities are judged, thus varies across trials. We should therefore consider the potential effect of context dependency (Tversky, 1977) when interpreting the dissimilarity judgments. The changes of context (“zooming in” on subclusters) can be construed as either an advantage or a disadvantage. On the one hand, having the subject consider a different subset on each trial promises to yield a deeper and higher-dimensional reflection of the mental representation. The fact that dissimilarities for a subcluster will be scaled (i.e., zooming in) is a desirable effect of context, which is accounted for by our scaling-to-match of the partial arrangements. On the other hand, more complex context effects, such as those producing intransitivity of dissimilarities [e.g., dissimilarity(X,A) < dissimilarity(X,B) < dissimilarity(X,C), but dissimilarity(X,C) < dissimilarity(X,A)] cannot be accommodated by estimating a single RDM as we do here. Rather the RDM would have to be modeled as context dependent. If such context effects are present for a given stimulus set, our approach will yield a compromise between the RDMs associated with the different contexts.

These considerations reflect the fundamental complexity of dissimilarity judgments and their dependency on the task (including the item set context, the nature of the judgments, time constraints, and other factors). Instead of looking for a single “right” way of obtaining dissimilarity judgments, we need to acknowledge task dependency. It is reassuring that we find high across-method reliability in the validation experiment. Although the three methods differ in terms of item set context (pairs, whole set, subsets) and in the way the judgments are communicated, the results are very similar here. This suggests a degree of task-independence of shape dissimilarity judgments. In general, however, we need to consider the particular task when interpreting and comparing dissimilarity judgment results.

### Inverse MDS from multiple partial arrangements

The concept of inverse MDS could be interpreted in a number of ways. First, trivially, it could be interpreted as measuring the distances of a low-dimensional, e.g., 2D, MDS arrangement. Second, it could be interpreted as finding the set of all dissimilarity matrices consistent with a given low-dimensional arrangement (De Leeuw and Groenen, 1997). Third, it could be interpreted as finding a dissimilarity matrix (or set of such matrices) that is simultaneously consistent with multiple low-dimensional arrangements of item subsets. This latter interpretation might be novel. It arises naturally from our practical problem here of inferring the underlying dissimilarity matrix from multiple partial arrangements.

**Figure 3:**
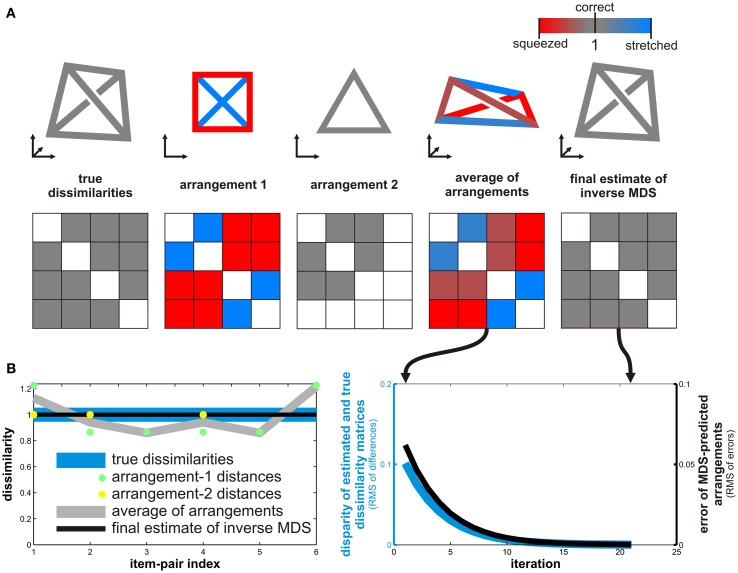
**Inverse MDS by iterative algorithm**. Inverse MDS estimates a higher-dimensional RDM from multiple low-dimensional arrangements. **(A)** In this toy example, we have four items and the dissimilarities are equal for all six pairs (all dissimilarities are 1), forming a tetrahedron in 3D (top left, RDM underneath in middle row). Arrangements are in 2D. We use MDS (optimizing metric stress) to simulate a subject’s arrangements. Arrangement 1 (second from left in top row) contains all four items. As the four items are optimally arranged in 2D, distances are squeezed and stretched (red and blue connections and RDM cells, respectively). Arrangement 2 (third from left in top row) contains only three of the four items. The three items’ RDM is perfectly represented by the 2D arrangement. A simple way to estimate the underlying RDM is to average the distance matrices of the 2D arrangements (second from right in top). Missing dissimilarity entries are ignored in the averaging and the matrices are first separately normalized by scaling the dissimilarities’ root mean square for the defined entries to the same value. **(B)** Results of iterative inverse MDS for the toy problem. The aligned-average RDM (gray line in left panel) is a better estimate of the true dissimilarities (blue line) than the distance matrix of arrangement 1 (green dots), but distortions remain. Inverse MDS can be performed by starting from the aligned-average matrix and iteratively improving the estimate. On each iteration, the current estimate is used to predict the subject’s arrangements by MDS. The disparities between the predicted and the actual arrangements are then used to adjust the estimate of the underlying RDM. The resulting iterative reduction of the error of the MDS-predicted arrangements (black line in right panel) reduces the disparity between the true and the estimated dissimilarity matrices (blue line in right panel; disparity measured as root mean square of differences after normalization of both matrices). In this toy example, the two arrangements shown suffice to perfectly recover the tetrahedral true RDM (black line in left panel).

A subject’s arrangements are always affected by placement error to some degree. Inferring the underlying RDM can be cast as an estimation problem. We described a procedure for improving an initial RDM estimate by iteratively driving down the error of the MDS-prediction of the subject’s arrangements. Although the algorithm works perfectly on the toy problem presented in Figure 3, it is not guaranteed to converge in general. Future studies might develop better methods for inverse MDS. For large numbers of items, however, inverse MDS poses a difficult optimization problem. The problem may also be ill-posed as the available arrangements may not provide sufficient constraints on the solution space. Nevertheless, an iterative reduction of the error of MDS-predicted arrangements will render the estimate more consistent with the arrangements that are available. The weighted-averaging approach provides a simple alternative that can be rapidly computed and may provide useful results in practice.

### Future directions

Our method appears to work well in practice even when the RDM is estimated simply by averaging the scaled-to-match partial RDMs. Our validation experiments here showed that the results are consistent with pairwise dissimilarity judgments and arrangement of pairs by similarity for an item set small enough for the latter two methods to be feasible. In another study (Mur et al., under review), we used the multi-arrangement method on a set of 96 object images, where the alternative methods would not have been feasible, because there are 4560 dissimilarities to estimate. The method worked well in this scenario, too, and the RDM from the judgments showed a substantial correlation with the RDM derived from brain representations of the object images. While the general approach of multi-arrangement appears promising, further validation is desirable and many aspects of our implementation here could be improved in the future.

#### Validity, reliability, and efficiency tests

For large item sets, free sorting and multi-arrangement are the only feasible methods for estimating a full RDM. We have discussed theoretical advantages of multi-arrangement, but it would be good to test empirically, which method has greater test-retest reliability and which method is more consistent with pairwise judgments (for smaller item sets for which the latter method is feasible). For small item sets, all methods are feasible. We have shown that the three methods used here yield reliable and consistent results. However, it is unclear which method should be preferred for a given number of items. It would be useful, thus, to empirically compare the efficiency with which different methods acquire RDM estimates. To this end, we could plot the test-retest RDM correlation for each method (vertical axis) against the time taken by the subject to do the task (horizontal axis). Such a comparison could be performed for various numbers of items. Pairwise judgments and arrangement of pairs by similarity would give points in the plot (one for each subject or averaged across subjects). For the multi-arrangement method, we could compute the test-retest RDM correlation (across sessions performed on different days) for multiple time periods using the RDM estimate obtained for all trials the subject completed in that period (e.g., after 10, 20, 30… min). The reliability of the RDM estimate could thus be plotted as a function of time. This approach would be useful also to test whether our adaptive trial-design heuristic provides a benefit over random subsets (Goldstone, 1994) or alternative trial-design methods.

#### Adaptive trial-design

Our “lift-the-weakest” adaptive item-subset design aims to optimize trial efficiency by constructing subsets that sample dissimilarities, for which we have the weakest evidence, and placing them in a small enough context of other items to enable the subject to “zoom in.” The focus on subclusters arises as a consequence of estimating the benefit and cost of presenting a given item-subset. The estimate of the trial benefit could be improved by using MDS to simulate the expected arrangement for the trial (using the current RDM estimate). Moreover, inverse MDS could form an integral component of item-subset design: We could determine the set of RDMs consistent with the arrangements thus far, and then design the next trial so as to optimally reduce the remaining uncertainty. This would take the distortions in the expected arrangements into account. In addition, the estimate of the trial cost (time taken) could be refined based on empirical evidence. Finally, an alternative approach to item-subset design would be to use explicit cluster analysis.

#### Inverse MDS

Inverse MDS poses a difficult and interesting challenge. Three directions of future development suggest themselves. (1) Optimization algorithm: Our iterative algorithm here represents only a first step. This local optimization approach could be run from multiple points to find global optima. Moreover, alternative global optimization techniques could be brought to bear on this problem. (2) Non-metric inverse MDS: In the present implementation, we interpret the distances in the subject’s arrangement as proportional to the internal representational dissimilarities. However, the subject might not achieve a proportional reflection of the distances in the internal representational space when arranging the objects. For example, if a subject attended preferentially to local relationships, her placements might not linearly reflect the internal representational space. Most fundamentally, the internal representational space might be inherently non-metric. These considerations motivate non-metric inverse MDS, in which we would use only the order of the distances in a given arrangement to estimate the RDM. (3) Explicit models of placement error: An explicit model of the arrangement errors could be integrated into the inverse MDS algorithm. This would enable us to take peculiarities of placement behavior into account. For example, subjects might avoid overlapping placement of two icons, even if the “true” dissimilarity demanded it. In general, placement error might not be Gaussian. An explicit model of placement error, motivated by empirical findings on placement behavior, might improve the RDM estimates.

## Conclusion

Practically, multi-arrangement provides an efficient solution to the problem of acquiring dissimilarity judgments. Theoretically, multi-arrangement poses the interesting problem of inverse MDS, constrained by multiple partial arrangements. Multi-arrangement is a generalization of pairwise judgments and free sorting. It reduces to pairwise judgments when only two items are presented on each trial. It reduces to free sorting when the subject is instructed to arrange the items into discrete piles. Pairwise judgment has the advantage of enabling independent continuous dissimilarity judgments and the disadvantages of taking a long time (quadratic in the number of items) and of deemphasizing the context of the item set. Free sorting has the advantage of being fast (linear in the number of items) and the disadvantage of providing only discrete same-different judgments for a given sorting. The optimal solution for a given application might fall in-between the special cases, suggesting that the space of multi-arrangement methods deserves to be explored.

## Conflict of Interest Statement

The authors declare that the research was conducted in the absence of any commercial or financial relationships that could be construed as a potential conflict of interest.

## Appendix

### Multi-arrangement by lift-the-weakest algorithm for adaptive design of item subsets

We present a particular implementation of the multi-arrangement method, which proceeds by the following algorithm:

1. trial 1: let subject arrange all items
2. estimate the RDM from the current set of item arrangements (see Estimating the High-Dimensional Dissimilarity Matrix from Multiple Item-Subset Arrangements for details)
3. if subject’s time is up or dissimilarity-evidence criterion reached, terminate
4. design item-subset for the next trial (optimizing trial efficiency)
5. next trial: let subject arrange the item-subset
6. goto 2

Steps 1 and 5 are carried out by an algorithm that presents the items on a computer screen and enables the subject to arrange them by mouse drag-and-drop operations. Step 2, the estimation of the RDM from a set of arrangements of subsets of the items (or “inverse MDS”), is detailed in Section “Estimating the High-Dimensional Dissimilarity Matrix from Multiple Item-Subset Arrangements.” Steps 3 and 4 are explained in this section.

The design of the item-subset to be presented on the next trial (step 4) is carried out by the “lift-the-weakest” algorithm. The motivation for the lift-the-weakest algorithm is to prioritize acquisition of evidence for those dissimilarities, for which we currently have the weakest evidence (thus the name “lift-the-weakest”). This prioritization lends the algorithm its “anytime” behavior (after the initial trial including all items has been completed).

More precisely, the lift-the-weakest algorithm heuristically optimizes the estimated *trial efficiency* for the subsequent trial, based on a few assumptions. Before we state the algorithm for item-subset design, we explain the assumptions and define the quantitative concepts involved:

- *Relative dissimilarities*: each item-subset arrangement is assumed to reflect the relative, not the absolute, dissimilarities between the items. A distance of 0 always represents identity. However, the scaling of the set of distances for a given arrangement is to be ignored. The subject is instructed to use the entire arena to arrange each subset. The same dissimilarity will therefore tend to correspond to a larger on-screen distance if the two items appear as part of a smaller subset.
- *On-screen placement error*: we assume that the location of each item in an arrangement is affected by placement error, which is isotropic and has a constant variance in on-screen distance units across trials. We further assume that the placement error is small relative to the distances.
- *Dissimilarity signal-to-noise ratio*: the dissimilarity signal is reflected in the on-screen distance. Since the placement error is constant in on-screen distance units, the dissimilarity signal-to-noise ratio is proportional to the on-screen distance. An item pair’s distance will therefore tend to reflect the dissimilarity (relative to the subset) with a higher signal-to-noise ratio if the two items are placed farther apart on a given trial (e.g., because there were fewer items in the trial allowing the subject to “zoom in” on a subcluster).
- *Dissimilarity-evidence weight = signal-to-noise ratio*^2^: the weight of the dissimilarity-evidence contributed by a given arrangement is the square of the signal-to-noise ratio. The dissimilarity for a given item pair can be estimated as an optimally weighted average of the scale-adjusted dissimilarity estimates from individual arrangements. According to the optimal-filter theorem, weighting each scale-adjusted dissimilarity estimate by the evidence weight gives the minimum-variance estimate of the dissimilarity (assuming independence of the evidence from different trials and neglecting distortions due to dimensionality reduction). An algorithm for such an estimate is described in Section “Inverse Multidimensional Scaling by Iterative Reduction of Arrangement Prediction Error,” below.
- *Dissimilarity-evidence matrix*: we can estimate the combined current evidence for the dissimilarity of each item pair by summing the evidence weights across trials. This yields a dissimilarity-evidence matrix (*n* by *n*, symmetric about an ignored diagonal), which is updated after each trial.
- *Termination when time is up or dissimilarity-evidence criterion is reached*: after each trial, we assess whether another trial should be performed. The process will be terminated when either the available time (e.g., 1 h) is up or a dissimilarity-evidence criterion is reached. The dissimilarity-evidence criterion is that the minimum current dissimilarity-evidence across all item pairs exceeds some threshold.
- *Lift-the-weakest*: the rationale of the algorithm is to seek more evidence for those item pairs, for which the current evidence is weakest. This is motivated by the idea that we want a minimum level of evidence for each item pair. Gathering evidence first for pairs for which we most lack it, ensures that the subject’s time is well spent. After the initial arrangement of all items, each additional trial improves the estimates, but the process can be terminated at any point. We call this “anytime” behavior. To formalize the goal to “lift-the-weakest,” we define the concept of “evidence-utility.”
- *Evidence-utility*: we would like a given amount of evidence to be considered more useful for an item pair, for which we have little dissimilarity-evidence so far, than for an item pair, for which we already have a lot of dissimilarity-evidence. We therefore define the evidence-utility *u* (i.e., the usefulness or value of the current evidence) as a saturating function of the current evidence weight *w* for an item pair:
  $$u\left(w\right)=1-e^{-w\cdot d},$$
  where *d* is the evidence-utility exponent, which controls how soon the utility saturates as a function of the evidence weight. The function *u*(*w*) starts at the origin with a positive slope and approaches an asymptote of *u *= 1. It models the notion that additional evidence for a given dissimilarity is less useful when we already have enough evidence for that dissimilarity. (In the current paper we used *d *= 10. The utility then saturates at an evidence weight of about 0.5. This focuses the item-subset design on lifting dissimilarities with an evidence weight below 0.5. The process was terminated when all dissimilarities had an evidence weight exceeding 0.5.) We compute an evidence-utility matrix (*n* by *n*, symmetric about an ignored diagonal), which assembles the evidence utilities for all item pairs.
- *Trial benefit = evidence-utility gain*: we define the benefit of having the subject arrange a particular item-subset as the evidence-utility gain, i.e., the change in total evidence-utility (summed across all item pairs) expected to occur if that trial were presented. To estimate this, we first consider the largest dissimilarity among the item set (based on the current dissimilarity estimate). We assume that the two items in this pair would end up at opposite ends of the arena, defining the scaling factor for the arrangement. The scaling factor enables us to estimate the on-screen distances and the expected signal-to-noise ratios, i.e., the expected dissimilarity-evidence the trial would contribute for each pair. This, in turn, enables us to estimate the current dissimilarity-evidence after the trial, and the associated evidence-utility matrix. The evidence-utility gain is the total evidence-utility after the trial minus the total evidence-utility before the trial.
- *Trial cost = time taken to arrange the subset*: we define the cost of the trial as the time it will take the subject to arrange the item-subset. We estimate this as a simple function of the number *n* of items in the trial: time = *n*^1.5^. The choice of 1.5 is motivated by the idea that an arrangement’s time complexity is superlinear, but subquadratic. The arrangement would take linear time if each item placement took constant time with no need to consider relationships. The arrangement would take quadratic time if each pairwise comparison were given an equal amount of time to adjust it. (The exponent or the form of the equation could be adjusted in the future on the basis of empirical measurements of the relationship between arrangement times and item set sizes.)
- *Trial efficiency = benefit/cost*: we define the trial efficiency, which we aim to optimize in designing each trial, as the trial benefit divided by the trial cost. The *t*rial *e*fficiency *TE* for an item-subset *ISS* is denoted *TE(ISS)* below. Note that *TE(ISS)* depends on the arrangements already performed, but this dependency is omitted from the notation for brevity [*TE(ISS)* = *TE(ISS*, arrangements already performed)].

#### Heuristic optimization of the estimated efficiency of the next trial

Based on the concepts introduced above, there is an item-subset that maximizes the estimated efficiency of the subsequent trial, given the current dissimilarity-evidence (i.e., the item-subset arrangements acquired so far). Because the number of item subsets to be considered is vast for larger item sets (2*^n^*; e.g., 2^40^ ≈ 10^12^ for 40 items), we do not search the entire set of subsets so as to find the global maximum of estimated trial efficiency. Instead we take a heuristic greedy approach. We start with the two items for whose dissimilarity we have the weakest evidence. We then consider adding each other item, and include the item (if any) that yields the highest positive gain in trial efficiency. This process is repeated until the point where adding any item will decrease the trial efficiency.

Here is the precise definition of the lift-the-weakest algorithm:

1. compute the current dissimilarity-evidence matrix (*n* by *n*)
2. find the item pair {*j*, *k*} with the weakest current dissimilarity-evidence
3. initialize the *i*tem-*s*ub*s*et for the *next* trial with those two items: *nextISS *= {*j*, *k*}
4. initialize the *cur*rent-*t*rial *e*fficiency: *curTE *= 0
5. find the *opt*imal *item* to add: *optItem* = argmax*_i_* (*TE(nextISS* ∪ {*i*})), where *i* ∈ total item set \ nextISS (if all items are in *nextISS* already, *optItem *= {}) and *TE*(item set) is the *t*rial *e*fficiency of an item set
6. if *TE(nextISS* ∪ {*optItem*}) ≤ *curTE*, then terminate, returning item-subset *nextISS*
7. add *optItem* to the next trial’s item-subset*: nextISS *= *nextISS* ∪ {*optItem*}
8. *curTE* = *TE(nextISS* ∪ {*optItem*})
9. goto 5

Because *curTE* is set to 0 initially, the item-subset will have at least three items. This is necessary in the context of the current implementation of the multi-arrangement, because the scaling of the distance matrix is to be ignored, rendering a two-item trial uninformative.

In this algorithm, the consideration of adding a particular item takes into account a number of consequences affecting the efficiency of the trial:

- If the item is added, the trial will produce evidence for the dissimilarities of the added item to all the other items already in the set. This increases the trial benefit.
- If the item is added, the trial will take longer to complete. This increases the trial cost.
- If the item is added, the on-screen distances of the arrangement might be scaled down, as the entire arrangement needs to fit into the arena. This would reduce the signal-to-noise ratios (i.e., the evidence) for the dissimilarities, thus reducing trial benefit.

The algorithm estimates these effects and chooses a trial with a good benefit-to-cost ratio, i.e., a highly efficient trial.

### Estimating the high-dimensional dissimilarity matrix from multiple item-subset arrangements

In this section we define the two particular algorithms for estimating the high-dimensional RDM from multiple item-subset arrangements (Figure 3).

#### Weighted average of iteratively scaled-to-match subset dissimilarity matrices

This algorithm is motivated by the assumption that each arrangement containing a given item pair provides an independent measurement of the dissimilarity for that item pair. The signal-to-noise ratio varies across the arrangements, because a given dissimilarity will not stand out from placement noise when it corresponds to a small on-screen distance in the context of a given arrangement. The dissimilarity for a given item pair can be estimated as a weighted average of the scale-adjusted dissimilarity estimates from individual arrangements.

We assume that placement error additively affects the dissimilarities, positively or negatively. (This holds if the placement error is small relative to the on-screen distance, in which case we can neglect the fact that placement noise cannot render distances negative.) According to the optimal-filter theorem, an average of the scale-adjusted dissimilarity estimates weighted by the evidence weights (squared signal-to-noise ratios) then gives the minimum-variance estimate of the dissimilarity for each item pair.

Because subjects are instructed to “zoom in” on subclusters in separate trials, we need to ignore the on-screen scale of each arrangement. We therefore determine a scaling factor for each subset RDM that matches the dissimilarities to an initial RDM estimate before weighted-averaging. The weighted average RDM estimate depends on the scaling factors. Conversely, the scaling factors are determined by the match to an initial RDM estimate. We therefore proceed iteratively, alternately adjusting the scaling factors and recomputing the weighted average RDM estimate until convergence. This is achieved by the following algorithm:

1. for each arrangement and each item pair included in it, determine the dissimilarity-evidence weight (as the squared on-screen distance)
2. determine the initial seed for the current RDM estimate
  - if a full arrangement (e.g., from the first trial) is included, use this as the current RDM estimate
  - otherwise, use the evidence-weighted average across arrangements of the on-screen distances as the current RDM estimate
3. scale the current RDM estimate to have a root mean square (RMS) of 1
4. scale the distance matrix of each arrangement, such that the RMS of its distances match the RMS of the same item pair entries of the current RDM estimate
5. replace the current RDM estimate with an evidence-weighted average of the scaled distance matrices. In other words, for each item pair: $\text{dissimilarity}\;\text{estimate}=\text{su}{\text{m}}_{ti}\left(\text{scaled}\;\text{distanc}{{\text{e}}_{\text{ti}}}^{*}{\text{unscaled distance}}_{ti}^{\text{2}}\right)∕\text{su}{\text{m}}_{ti}\left({\text{unscaled distance}}_{ti}^{\text{2}}\right),$ where *ti* is the *t*rial *i*ndex running through all trials that included the item pair
6. if the RMS of the deviations between the current and the previous RDM estimate is close to 0, return the current RDM estimate
7. otherwise, goto 3

#### Inverse multidimensional scaling by iterative reduction of arrangement prediction error

The weighted-averaging algorithm explained above (Inverse Multidimensional Scaling by Iterative Reduction of Arrangement Prediction Error) neglects distortions due to the projection into the 2D arena. We use the term “inverse MDS” for more ambitious methods that take the distortions due to dimensionality reduction into account. We can estimate a (high-dimensional) RDM from multiple (low-dimensional) arrangements by predicting the arrangements from an initial estimate of the RDM, and iteratively reducing the prediction errors. The following algorithm implements this approach:

1. compute an initial estimate of the RDM using weighted-averaging of subset RDMs (as described in Weighted Average of Iteratively Scaled-to-Match Subset Dissimilarity Matrices)
2. scale the current RDM estimate to an RMS of 1
3. for each arrangement with trial index *ti*
  - reduce the current RDM estimate to the subset of items arranged in trial *ti*
  - use MDS (with metric stress criterion) to predict the subject’s arrangement
  - compute the distance matrix from the MDS-predicted arrangement
  - scale the distance matrix for the *predicted* arrangement to match its RMS to the RMS of the corresponding dissimilarities in the current RDM estimate
  - scale the distance matrix for the *actual* arrangement to match its RMS to the RMS of the corresponding dissimilarities in the current RDM estimate
  - compute the dissimilarity disparities D*_ti_* by subtracting the scaled distance matrix for the *actual* arrangement from the scaled distance matrix for the *predicted* arrangement
4. compute the RMS of the disparities D*_ti_* across item pairs and trial indices
5. if the RMS of the disparities is below a threshold, return the current RDM estimate
6. for each item pair *ip*, compute the dissimilarity adjustment A*_ip_* as the average of the dissimilarity disparities D*_ti_*
7. adjust the current RDM estimate by subtracting the dissimilarity adjustments c*A*_ip_*, where c is a constant scaling factor (c = 0.3 in our code)
8. set any dissimilarities that are negative after adjustment to 0 in the current RDM estimate
9. goto 2

This method perfectly recovers the true underlying dissimilarities for the toy example shown in Figure 3. It significantly improves RDM estimates for more complex scenarios. However, if the item set is large (e.g., *n* = 100 items), the inversion is a difficult problem because of the large number of parameters of the RDM [(*n*^2^ − *n*)/2 = (100^2^ − 100)/2 = 4950 dissimilarities]. In that case, compared to the weighted-averaging approach, this method will still improve the RDM estimate in the sense that the result will be more consistent with the arrangements. However, convergence to an accurate estimate of the actual underlying dissimilarities is not guaranteed, as it will depend on the quantity and quality of the arrangement data. Although multiple arrangements help to constrain the space of solutions, a unique solution may not exist.
